# Short-Term Lincomycin Exposure Depletion of Murine Microbiota Affects Short-Chain Fatty Acids and Intestinal Morphology and Immunity

**DOI:** 10.3390/antibiotics9120907

**Published:** 2020-12-14

**Authors:** Shunfen Zhang, Ruqing Zhong, Hui Han, Bao Yi, Jie Yin, Liang Chen, Hongfu Zhang

**Affiliations:** 1 State Key Laboratory of Animal Nutrition, Institute of Animal Science, Chinese Academy of Agricultural Sciences, Beijing 100193, China; 82101182376@caas.cn (S.Z.); zhongruqing@caas.cn (R.Z.); hanhui16@mails.ucas.ac.cn (H.H.); yibao@caas.cn (B.Y.); zhanghongfu@caas.cn (H.Z.); 2 College of Animal Science and Technology, Hunan Agricultural University, Changsha 410128, China

**Keywords:** antibiotic, microbiota, metabolites, TLR, intestinal morphology

## Abstract

Lincomycin, as one of the most commonly used antibiotics, may cause intestinal injury, enteritis and other side effects, but it remains unknown whether these effects are associated with microbial changes and the effects of different doses of lincomycin on infants. Here, 21-day old mice were exposed to 1 and 5 g/L lincomycin to explore the effects of lincomycin on the gut microbiota, metabolites and inflammation. Compared to the control mice, 1 g/L lincomycin exposure decreased the body weight gain of mice (*p* < 0.05). Both 1 and 5 g/L lincomycin exposure reduced the diversity and microbial composition of mice (*p <* 0.05). Furthermore, 1 and 5 g/L lincomycin reduced the relative concentrations of acetate, propionate, butyrate, valerate, isobutyric acid and isovaleric acid in the colon chyme of mice (*p <* 0.05). In addition, 5 g/L lincomycin exposure reduced the villus height, crypt depth, and relative expression of *TLR2*, *TLR3*, *TLR4*, *IL-18*, *TNF-α*, and *p65* in the jejunum of mice (*p* < 0.05), while 1 g/L lincomycin exposure reduced the relative expression of *TLR2*, *TLR3*, *TNF-α*, and *p65* (*p <* 0.05). Collectively, these results highlight the depletion effect of short-term lincomycin exposure on microbiota and the further regulatory effect on intestinal morphology and immunosuppression in infant mice.

## 1. Introduction

Over the past decades, antibiotics have made a significant contribution to public health and are also widely used in animal husbandry to promote growth, but antibiotic resistance is causing people to rethink and restrict the use of antibiotics. Recently, the effect of antibiotics on human intestinal health and immunity, especially the health of young children, has become a hot topic. Gut microbiota which is abundant in the gastrointestinal tract plays a key role in host health, and is also implicated in the pathogenesis of several diseases (obesity, diabetes, autoimmunity, and malignancies especially colorectal cancer) [1]. It is well-documented that gut microbiota can break down food and promote the utilization of nutrients [2]. Beyond that, the gut microbiota also protects the host by adjusting immunity and inhibiting pathogenic bacteria [3]. They may directly or indirectly produce short-chain fatty acids (SCFAs) [4]. Antibiotics, which work by killing or suppressing pathogens, also disrupt symbiotic bacteria and may induce pseudomembranous colitis. But because of the various types and doses, the antibiotics may have differing effects on microbiota and host [5]. For example, ampicillin, vancomycin, ciprofloxacin, and metronidazole treatment could significantly suppress the production of SCFAs and increased total systemic immunoglobulin E production in mice [6]. Another study showed that enrofloxacin, cephalexin, paromomycin, and clindamycin exposure consumed the colonic microbiota and caused mucosal inflammation in rhesus macaques [7]. However, to achieve the therapeutic effect more quickly, the overuse of antibiotics during infection is common. There are growing concerns that antibiotic treatment may lead to long-term negative health impact [8]. Thus, it is urgent to better understand the complex consequences of commonly used antibiotic treatment.

Lincomycin is commonly used to treat a variety of Gram-positive bacterial infections, including respiratory tract, skin, and soft tissue infections [9]. Due to its low price and no need for a skin test, lincomycin is widely used in clinical practice. Besides, lincomycin is often used as a substitute for penicillin, and its side effects are mainly gastrointestinal reactions. It is also widely used to treat diseases such as diarrhea and fever in animal husbandry. Long-term use of lincomycin has been shown to cause diarrhea, inflammatory bowel disease, and pseudomembranous colitis [10]. These consequences may be related to changes in gut microbiota. However, the mechanisms of these effects remain to be further explored. And there is less attention received on the effects of short-term lincomycin exposure on the infant host’s gut microbiota, intestinal health, and inflammation.

Here, this study aimed to determine the effects of different doses of lincomycin on gut microbiota in infant mice, as well as changes in metabolites, intestinal morphology, and immune function in mice. In the present study, ICR mice were administered 1 and 5 g/L lincomycin for 1 week, respectively, to determine changes in gut microbiota, intestinal morphology, and the immune response to lincomycin.

## 2. Results

### 2.1. Antibiotic Exposure Can Decrease Body Weight Gain of Mice

**Figure 1:**
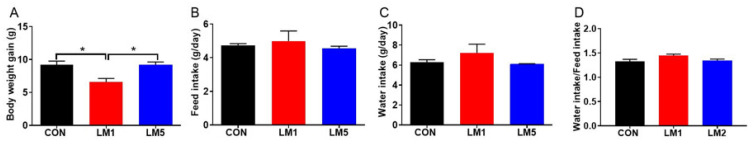
Lincomycin reduces growth performance of mice. (**A**) Body weight gain in each group of mice. (**B**) Feed intake in each group of mice. (**C**) Water intake in each group of mice. (**D**) Water intake/feed intake in each group of mice. Values are means (n 10/diet), with their standard errors represented by vertical bars. Statistical analysis was performed using one-way ANOVA with multiple comparisons by Fisher LSD test. * Means statistically significant (*p* < 0.05).

In order to be more in line with the reality in the field of human medicine and animal husbandry, especially in developing countries, we have chosen two different doses of lincomycin to explore their impact on feed and water intake, and body weight in mice. We fed 21-day old mice with 1 g/L lincomycin (LM1) and 5 g/L lincomycin (LM5) in drinking-water for 7 days, and control mice were fed with drinking-water (CON) for 7 days. The results showed that 1 g/L lincomycin significantly reduced the body weight gain of the mice (*p <* 0.05), but 5 g/L lincomycin had no effects on body weight gain (Figure 1A). The treatments of 1 g/L and 5 g/L lincomycin exposure had no effects on feed intake, water intake, and mice (Figure 1B–D). Altogether, these findings demonstrated that lincomycin had negative effects on the body weight gain of mice.

### 2.2. Antibiotic Treatment Reduces the Amount and Variability of the Gut Microbiota

**Figure 2:**
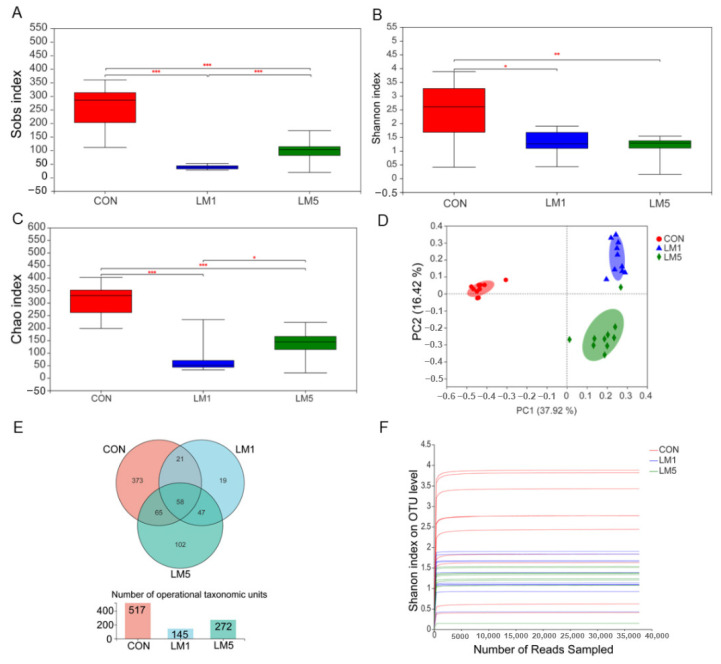
Microbial community composition of mice. (**A**) The alpha-diversity of fecal microbiota in colon chyme of Sobs index. (**B**) The alpha-diversity of fecal microbiota in colon chyme of Shannon index. (**C**) The alpha-diversity of fecal microbiota in colon chyme of Chao index. (**D**) Weighted principal coordinate analysis (PCoA). (**E**) Venn diagram. (**F**) Rarefaction curves tended to reach the plateau. * *p* < 0.05; ** *p* < 0.01; *** *p* < 0.001.

To determine the impact of lincomycin on gut microbiota, we measured bacterial populations in colon chyme samples for all groups by 16S rRNA gene sequencing. After removing unqualified sequences following the previously reported method, the 30 samples from three groups produced a total of 1,505,868 and an average of 50,195 ± 1242 effective reads. The rarefaction curves indicated that the sequencing was deep enough to capture most of the operational units within our samples (Figure 2F). Principal coordinate analysis (PCoA) based on the unweighted unifrac distance metric is shown in Figure 2D. There was a strong impact of lincomycin on the beta diversity of the gut microbiota in mice. Alpha diversity (Sobs, Shannon, and Chao index) also revealed the gut microbial flora diversity of lincomycin-treated mice was significantly lower compared with control mice (Figure 2A–C, *p* < 0.01). Furthermore, the Sobs and Chao index in LM5 were greater than that in LM1 (*p* < 0.05). There were 517, 145, and 272 operational taxonomic units (OTUs) obtained from CON, LM1, and LM5 mice, respectively, of which 58 were common OTUs among the three experimental groups (Figure 2E). Moreover, 373 unique OTUs were found in CON, but only 19 and 102 unique OTUs in LM1 and LM5, respectively.

**Figure 3:**
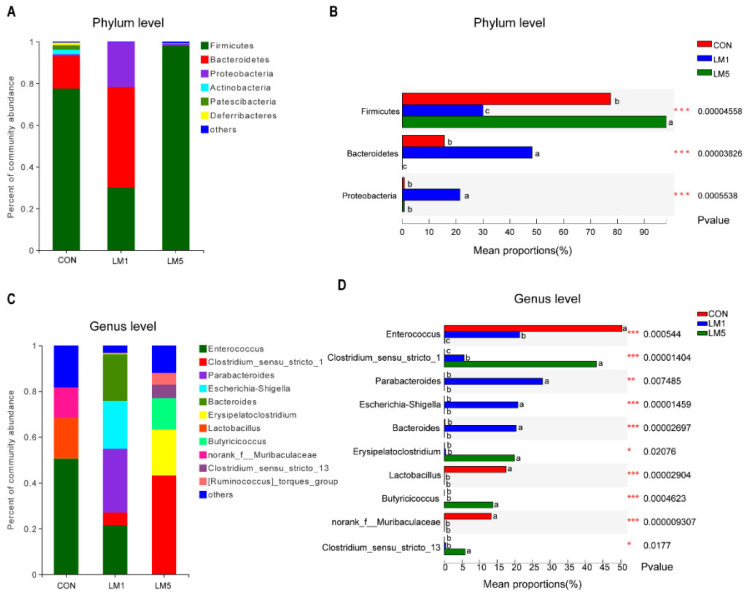
Influence of lincomycin treatment on the microbial at the phylum and genus levels in mice. (**A**) The relative abundance of bacterial at the phylum level. (**B**) The top three bacterial at the phylum level statistical comparison of the relative abundance in three groups. (**C**) The relative abundance of bacterial at genus levels. (**D**) The top ten bacterial at the genus level statistical comparison of the relative abundance in three groups. Less than 1% abundance of the phyla or genus was merged into others. Different letters indicate significant differences between the two groups, while the same letter indicates insignificant differences. * *p* < 0.05; ** *p* < 0.01; *** *p* < 0.001.

Notably, the microbial community composition at the phylum level of three groups was shown in Figure 3A. The composition of the gut microbiota of control mice at the phylum level included six major phyla, *Firmicutes*, *Bacteroidetes*, *Actinobacteria*, *Proteobacteria*, *Patescibacteria*, and *Deferribacteres*. The relative abundance of the phyla *Firmicutes*, *Actinobacteria*, and *Deferribacteres* was decreased, while the relative abundance of the phyla *Bacteroidetes* and *Proteobacteria* was increased for 1 g/L lincomycin administration group. However, a dramatic decreased in the abundance of the phyla was observed at 5 g/L lincomycin group, and 99% of them were *Firmicutes*. It is usually considered that a high relative abundance of *Proteobacteria* and *Bacteroidetes* are not beneficial to health [11]. Compared to the CON group, the proportion of *Bacteroidetes* and *Proteobacteria* was increased in LM1 (*p <* 0.05, Figure 3B). However, the *Bacteroidetes* were significantly decreased in LM5 (*p <* 0.05).

At the genus level, the composition of the gut microbiota in control mice included 3 major genera, *Enterococcus*, *Lactobacillus*, and *norank_f__Muribaculaceae* (Figure 3C). The *Enterococcus, Parabacteroides*, *Escherichia-Shigella*, *Bacteroides*, and *Erysipelatoclostridium* were the primary genera in LM1. Interestingly, we found *Clostridium_sensu_stricto_1*, *Butyricicoccus*, *Clostridium_sensu_stricto_13*, and *Erysipelatoclostridium* were abundance in LM5. Next, we compared the major bacterial genera among the three groups (Figure 3D). The abundances of *Enterococcus*, *Lactobacillus*, and *norank_f__Muribaculaceae* were significantly reduced in LM1 and LM5 compared with CON (*p < 0.05*). However, the relative abundance of *Parabacteroides*, *Escherichia-Shigella*, and *Bacteroides* in LM1 were significantly increased in CON and LM5 (*p <* 0.05). The relative abundance of *Clostridium_sensu_stricto_13*, *Erysipelatoclostridium*, *Butyricicoccus,* and *Clostridium_sensu_stricto_1* in LM5 were greater than CON and LM1 (*p <* 0.05). All these results showed that lincomycin exposure reduced the number of intestinal microbiota and affected the composition of microbiota in mice, while 1 and 5 g/L lincomycin had different effects on intestinal microbiota in mice.

### 2.3. Effects of Antibiotic on SCFAs Production

**Figure 4:**
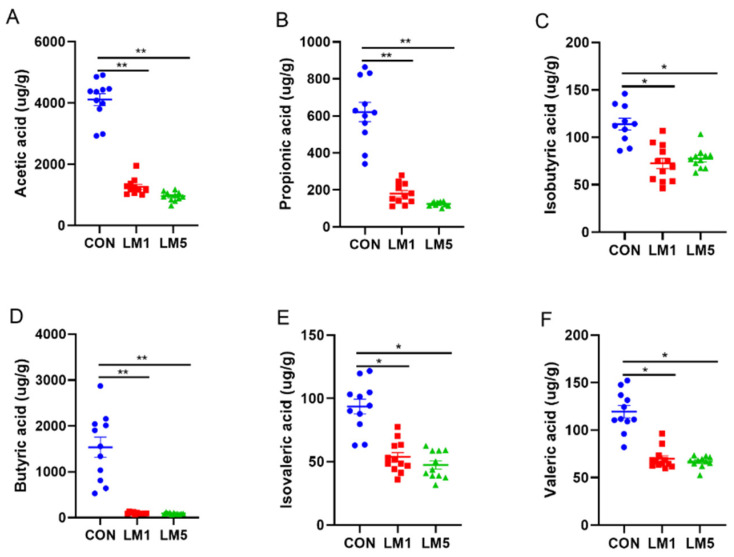
Concentrations of short-chain fatty acids (SCFA) in fresh colonic chyme after treated with lincomycin in mice. Scatter plot of SCFA concentration. Statistical analysis was performed using one-way ANOVA with multiple comparisons by Fisher LSD test. * *p* < 0.05; ** *p* < 0.01. (**A**) Acetic acid. (**B**) Propionic acid. (**C**) Isobutyric acid. (**D**) Butyric acid. (**E**) Isovaleric acid. (**F**) Valeric acid.

To evaluate the effects of lincomycin on the metabolites of the intestinal microbiota, we examined the relative concentration of SCFAs in colon chyme (Figure 4). The results showed that the relative concentration of acetate, propionate, butyrate, valerate, isobutyric acid, and isovaleric acid was dramatically reduced both in LM1 and LM5 compared with CON (*p <* 0.05). And the relative concentration of acetate, propionate, butyrate, valerate, isobutyric acid, and isovaleric acid were no difference between LM1 and LM5. These results indicated that lincomycin had a negative effect on the concentrations of SCFAs in mice.

### 2.4. Antibiotic Exposure Influence Morphology of Jejunum and Ileum and Intestinal Barrier/gut integrity in Mice

**Figure 5:**
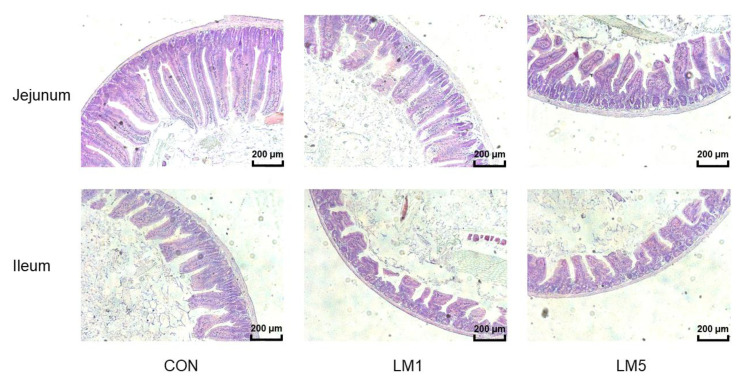
To evaluate the potential intestinal tissue injury caused by antibiotic, the staining profiles by H&E of jejunum and ileum in mice of three groups were displayed (*n* = 10/group). Scale bars: 100 μm.

The intestinal morphology of mice was presented in Table 1 and Figure 5. The jejunum and ileum of the mice in CON were integral, and composed of slender villi and complete crypts, which include arranged neatly epithelia cell. On the contrary, in LM1 and LM5, the villi were short and small in number, the crypts are irregular and shortened. Besides, there were some shed villi in lumens, and the epithelial cells were irregular and disorganized. In the jejunum and ileum, villus height and crypt depth of the mice in LM5 was reduced compared with CON (*p* < 0.05). Villus height and crypt depth of the mice in LM1 was below CON and above LM5 statistically, but not to a significant level. The V/C ratio both in jejunum and ileum were similar in three groups. These results declared that lincomycin had negative effects on the intestinal morphology and intestinal barrier of mice, and the effect increases with the dosage.

**Figure 6:**
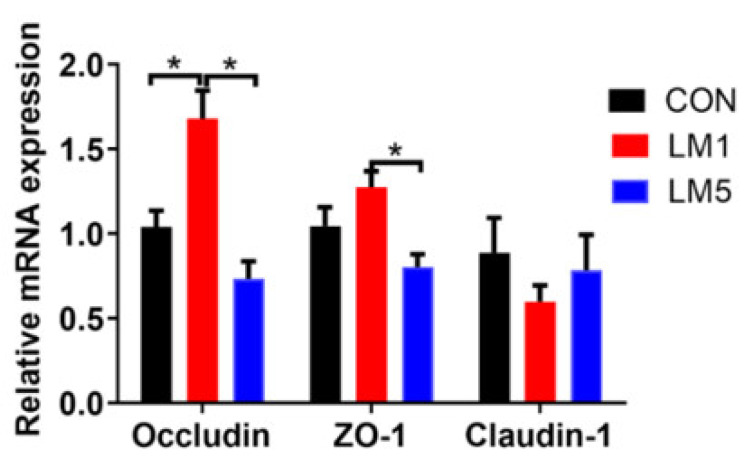
Effect of lincomycin on intestinal barrier in mice jejunal, relative expression of *ZO-1*, *Occludin*, and *Claudin-1* mRNA. Values are means, with their standard errors represented by vertical bars. Statistical analysis was performed using one-way ANOVA with multiple comparisons by Fisher LSD test, * *p* < 0.05.

To evaluate the effects of lincomycin on intestinal barrier/gut integrity, we determined the mRNA expressions of the tight junction components *cytosolic scaffold proteins (ZO-1)*, *Occludin*, and *Claudin-1* in the jejunum (Figure 6). The results showed that the expression of *Occludin* in LM1 was improved compared with the CON and LM5 (*p <* 0.05), but there was no difference between LM5 and CON (*p >* 0.05). Moreover, the expression of *ZO-1* in LM1 was greater than LM5 (*p <* 0.05), but both 1 and 5 g/L lincomycin had no effects on the expression of *ZO-1* and *Claudin-1* (*p >* 0.05). These results declared that 1 and 5 g/L lincomycin-induced intestinal dysbiosis may have a different effect on gut permeability.

### 2.5. Effect of Antibiotic on TLRs Expression Patterns and Inflammation of Mice

**Figure 7:**
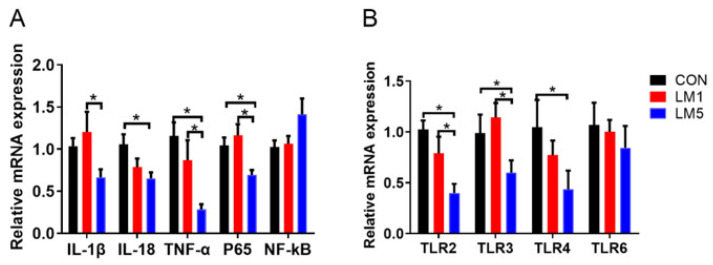
Effect of lincomycin on inflammatory cytokine expression in mice jejunal. (**A**) Relative expression of *IL-1β*, *IL-18*, *TNF-α*, *P65*, and *NF-κB* mRNA in the jejunum of mice. (**B**) Relative expression of *TLR2*, *TLR3*, *TLR4*, and *TLR6* mRNA in the jejunum of mice. Values are means, with their standard errors represented by vertical bars. Statistical analysis was performed using one-way ANOVA with multiple comparisons by Fisher LSD test, * *p* < 0.05.

To examine whether lincomycin induces inflammation in mice, the mRNA levels of the inflammatory cytokine *TNF-α*, *IL-1β*, and *IL-18* in jejunum were determined (Figure 7A). Compared with CON, the expression of *IL-1β*, *IL-18*, and *TNF-α* in the jejunum was decreased in LM5 (*p <* 0.05). The expression of *IL-1β* and *TNF-α* in LM5 of the jejunum was decreased compared with LM1 (*p* < 0.05). However, the expression of *IL-1β*, *IL-18*, and *TNF-α* have no difference between LM1 and CON.

In order to investigate the mechanism of lincomycin on the intestinal inflammatory of mice, *TLR2*, *TLR3*, *TLR4*, *TLR6*, as well as *NF-κB* signaling pathways were examined. Figure 7B shows different TLR expression in the jejunum of mice treated with lincomycin. The treatment of 5 g/L lincomycin reduced the expression of *TLR2, TLR3*, and *TLR4* in the jejunum (*p <* 0.05) but had no effects on *TLR6* in the jejunum. The treatment of 1 g/L lincomycin treatment had no effects on *TLR2*, *TLR3*, *TLR4*, and *TLR6* expression in jejunum compared with CON, while the expression of *TLR2* and *TLR3* was greater in LM1 than LM5 (*p <* 0.05). Compared with CON, the expression of *P65* in LM1 and LM5 was reduced (Figure 7A, *p <* 0.05). However, the expression of *NF-κB* had no differences among three groups (Figure 7A).

### 2.6. Antibiotic Modulates the Relevance Network between Relative Expression of Cytokine Contents, Bacterial Members, and Metabolites

**Figure 8:**
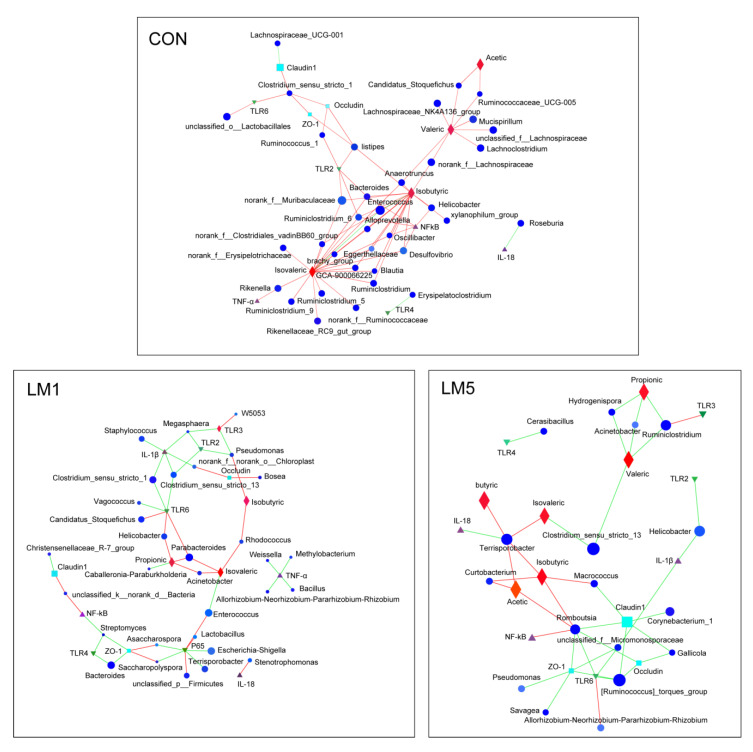
A network for correlation analysis among the relative abundances of microbiota phylum, the concentrations of SCFAs, and the relative expression of cytokines in the mice of CON, LM1, and LM5, respectively. Only correlations with a partial spearman’s coefficient >0.5 and a *p* < 0.05 were shown. The different color and shapes of nodes indicates various targets as described in legend, and the size of nodes indicates species abundance. The line color represents correlation, red represents positive correlation and green represents negative correlation.

To explore the interaction among microbiota, SCFAs, and cytokines in the mice of three groups, these targets were used to construct a network for correlation analysis (Figure 8). The results showed that unique topological patterns were observed among groups. For CON, a large number of bacterial genera formed a positively regular network centered on isobutyric, isovaleric, and valeric acid, suggesting the contribution of plentiful symbiotic bacteria to intestinal homeostasis. However, for LM1 and LM2 incompact and irregular networks emerged, in which the most relationships between microbiota and SCFAs/cytokines were negative, indicating that antibiotics disturbed the intestinal homeostasis in terms of microbiota, tight junctions, immune and metabolism.

## 3. Discussion

In this study, we found gut microbiota dysbiosis, immunosuppression, and intestinal barrier disruption in mice after a week of antibiotic exposure. Antibiotic exposure has a far-reaching impact on intestinal microbiota [12]. Manuzak et al. [7] reported that enrofloxacin, cephalexin, paromomycin, and clindamycin all disrupted the microbiome of rhesus macaques by reducing abundances of fermentative bacteria and increased abundances of potentially pathogenic bacteria such as *Enterobacteriaceae* in faeces, and decreasing *Helicobacteraceae* in the colon. In this study, we found the alpha diversity (Sobs, Shannon, and Chao index) of colon microbiota were significantly reduced by lincomycin exposure, and the microbial population were depleted. Specifically, the abundance of *Enterococcus, Lactobacillus,* and *norank_f__Muribaculaceae* were significantly reduced by lincomycin exposure, but more pathogens (*Parabacteroides*, *Escherichia-Shigella*, *Bacteroides*, and *Erysipelatoclostridium*) obviously grew in lincomycin treated mice. *Enterococcus* and *Lactobacillus* were thought to regulate intestinal flora and host immune function [13]. *Parabacteroides* have been reported to associate with abdominal infection [14]. While *Escherichia* and *Bacteroides* have been confirmed to induce chronic infection, IBD, and even colorectal cancer [15,16]. These results suggest that antibiotics disrupted intestinal microbiota and may produce complex responses.

In the present study, the abundance and number of microbiotas dramatically decreased in mice that exposure to 1 and 5 g/L lincomycin, and resulted in many adverse effects. In terms of weight, compared with CON, 1 g/L lincomycin exposure reduced the body weight gain of the mice, but 5 g/L lincomycin had no effects on the body weight gain of the mice. It takes energy to ferment pathogens in the gut, and it triggers an inflammatory response that the host needs energy to fight. Thus, these results seem to be due to the energy loss caused by lincomycin-induced decreased fermentation of hindgut microbiota in LM1. However, immunosuppression caused by high doses of lincomycin may reduce energy loss in LM5. Moreover, differences in nutrient absorption due to intestinal permeability and intestinal wall thickness may also affect body weight. In addition, these results may also be attributed to the lower level of *Firmicutes* and high levels of *Bacteroidetes*, which were thought to be associated with obesity [17]. In obese human subjects, fewer *Bacteroidetes* and more *Firmicutes* were characteristic of the intestinal microbiota [18]. A recent study indicated that *Bacteroidetes* had a positive correlation with LDL-and HDL-cholesterol levels, whereas *Firmicutes* had a negative correlation with total cholesterol, LDL-and HDL-cholesterol [19]. These results demonstrated that alternation of lipid metabolism may be another way in which gut microbes influence weight change. In the current experiment, the *Firmicutes* was reduced and the *Bacteroidetes* was improved in LM1, a low proportion of *Firmicutes*/*Bacteroidetes* caused a reduction of weight gain. However, a high level of *Firmicutes* and exhausted *Bacteroidetes* were observed in LM5, thus the body weight gain was unchanged. The specific mechanism of this result deserves further investigation.

SCFA is an important metabolite in the intestinal microbial environment and plays an important role in reducing inflammation and promoting intestinal health [20]. They are also closely involved in immune, anti-tumor, and anti-inflammatory activities [21]. In this study, we investigated gut microbial metabolites and found lincomycin exposure reduced the concentration of acetate, propionate, butyrate, valerate, isobutyric acid, and isovaleric acid in the colon. These results may be since lincomycin killed many SCFA-producing bacteria in the intestinal and led to the reduction of SCFA production which has also been seen in the study of Romick et al. [22]. For instance, *Lactobacillus,* which is depleted in LM1 and LM5, is acetic-producing bacteria. Furthermore, *Bacteroides*, *Butyricicoccus*, *Clostridium*, *Ruminococcus*, and *Ruminococcus* have been reported as SCFA-producing bacteria [23,24]. The relative abundance of *Bacteroides* in LM1 was more abundant than LM5 and CON, the abundance of *Butyricicoccus* and *Ruminococcus* were less than LM5, while the concentration of SCFA in LM1 and LM5 were similar in this study. These results may be due to a significant decrease of main SCFA-producing bacteria in *Firmicutes* and *Bacteroidetes*, which results in a low level of SCFAs concentration both in LM1 and LM5. In the correlation network analysis, we found that the abundance of *Rikenella, Ruminococcacea, Blautia,* and *Ruminiclostridium* were positively correlated with the isovaleric content, the abundance of *Anaerotruncus, Helicobacter, Bacteroides, Enterococcus,* and *Desuifovibrio* were positively correlated with the isobutyric content, and the abundance of *Lachnoclostridium, Mucispirillum, Lachnospiraceae,* and *Candidatus_Stoquefichus* were positively correlated with valeric content. However, in LM1 and LM5, we found the abundance of these microbiota were decreased. Meanwhile, the increased abundance of *Hydrogenispora, Acinetobacter,* and *Ruminiciostridium* in LM5 were observed to negatively correlate with propionic and valeric acid content. Therefore, the variation of these microbiota caused by antibiotic may be accompanied by a decrease in propionic, isovaleric, isobutyric, and valeric acid content. All of these results illustrated that antibiotic-induced microbial degradation may affect the production of SCFAs.

Intestinal symbiotic microbiota plays a key role in maintaining healthy intestinal barriers, which are very vital for resistance to infection by pathogenic microorganisms in the lumen [25]. *Bifidobacterium* has been reported to benefit intestinal homeostasis via competing for nutrients and adhesion sites with pathogens [26]. Antibiotics-induced symbiotic bacteria loss may reduce epithelial exposure with bacteria, followed by disturbing intestinal barrier function and integrity [27]. The downregulation of villus height and crypt depth in the jejunal and ileum (Table 1) suggested that lincomycin exposure may decrease the integrity of the mucous layer, which represented a vulnerable gut environment. It was reported that SCFAs, the substrate for the energy metabolism of intestinal epithelial cells, regulated the functional integrity of intestinal epithelial cells [28,29]. For instance, butyric acid has been found to maintain intestinal integrity by promoting epithelial energy metabolism [30]. Therefore, the low SCFAs in LM1 and LM5 may also contribute to reshaping intestinal morphology. The effects of lincomycin on the mRNA expression of various intestinal tight junction proteins which including occludin, claudin-1, and ZO-1 were also investigated in this study. We found the expression of *occludin* and *ZO-1* in LM1 were greater than LM5. The network analysis showed that the abundance of *Macrococcus*, *Corynebecterium-1*, *Romboutsia*, and *Gallicola* were negatively affected *claudin-1* and *occludin* expression, and the abundance of *Romboutsia, Psoudomonas, Savages,* and *Micromonosporacese* were negatively affected *ZO-1* expression in LM5. In addition, Li et al. [31] have reported that the reduced *Firmicutes*/*Bacteroidetes* ratio was along with augmented tight junction proteins in mice. Thus, the lincomycin-induced variation of *Firmicutes* and *Bacteroidetes* in LM1 could be closely associated with the intestinal tight junction barrier in this study. Increased mRNA levels of *ZO-1* and *occludin* in LM1 indicated the reduced intestinal permeability, which may hinder nutrient absorption in the short term. This may also be the reason why the body weight gain of mice in LM1 was less than CON and LM5. The results described above indicate that antibiotic-induced microbiota and SCFAs reduction may disrupt intestinal morphology and barriers.

Toll-like receptors (TLRs) are pattern recognition receptors (PRRs) that mediate the interaction between microbiota and the host, and very vital for innating immune responses [32,33]. In this experiment, lincomycin exposure consumed vast microbiota in the colon and changed the composition of the gut microbiota. Meanwhile, decreased expressions of *TLR2*, *TLR3*, and *TLR4* were observed after lincomycin exposure, indicating that the host-microbiota interactions might be also altered. Some symbiotic bacteria such as *Lactobacillus*, *Bifidobacterium* and *Enterococcus* have been reported to improve intestinal mucosal inflammation by regulating TLRs expression [34]. Thus, a possible reason for the decreased expressions of TLRs might be that the depletion of intestinal symbiotic microbiota caused by antibiotics reduces the stimulation of TLRs by symbiotic bacteria. These results were similar to the previous study that the combination of antibiotics ampicillin, vancomycin, neomycin, and metronidazole induced intestinal microbiota depletion decreased the *TLR4* expression of the intestinal mucosa in mice [35]. Furrie et al. [33] have reported that *Enterococcus* was positively correlated with *TLR4* expression. The depletion of *Enterococcus* in LM5 may be responsible for the reduction of *TLR4* in this study. Furthermore, TLR2 and TLR4 protein has been shown only in the crypts of mouse colon [36]. Therefore, lincomycin-induced crypts damage in LM5 may be another reason to reduce the expression of *TLR2* and *TLR4*. *TLR3* expresses at the luminal surface, and the expression may be enhanced by the interaction of new epithelial cells with gram-positive commensal organisms [33]. In this study, 5 g/L lincomycin treatment consumed gram-positive commensal organisms, leading to the decrease of *TLR3* expression. These results suggest that microbial depletion caused by antibiotics may affect TLR-related immune responses.

In general, TLRs play immunomodulatory role via activating NF-κB to initiate the release of inflammatory cytokines such as TNF-α and IL-1β through myeloid differentiation factor 88 (MyD88) dependent/independent pathway [37,38]. As communicators between immune cells, cytokines were crucial parts of the immune system [39]. Their changes were associated with host inflammatory responses. In this study, with the decrease of *TLR2* and *TLR4* expression, the expression of *TNF-α*, *IL-1β,* and *P65* was decreased in LM5. This result was similar to the study of Jena et al. [40] who reported that the ileal expression of gene *IL-1β* and *IL-6* were decreased in a polymixin B-treated mouse model. Previous studies have shown that gut microbiota interacts with the immune system by releasing cytokines [5]. For instance, TNF-α production may be related to *Odoribacter splanchnic* and the genus *Bilophila* [41]. The above results of this study may be related to the abundance of *Clostridium*, which induces Treg activity and inflammation [42]. In addition, in LM5, we also observed a decrease of *IL-18* expression in the jejunum which was vital in maintaining intestinal homeostasis. Meanwhile, the presence of *Terrisorobacter* and its negative correlation with IL-18 were also found in LM5. On the one hand, the reduction of microbial diversity caused by antibiotics reduces the stimulation of microorganisms to the host and the interaction of microbial-related molecular patterns with the host in the short term, thus reducing the inflammatory response. On the other hand, microbial metabolites had a strong regulation on cytokine formation [43]. Thus, the decrease of cytokines such as *IL-1β, TNF-α,* and *IL-18* expression in this study may be associated with the drastic reduction of acetate, propionate, butyrate, valerate, isobutyric acid, and isovaleric acid. These results suggest that antibiotics-induced microbial depletion may cause cytokine changes.

Lincomycin and related antibiotics, such as clindamycin, are widely used as prophylactic treatment in gastrointestinal operations, including colorectal resection for colorectal cancer. The reduction of beneficial SCFA along with the down-regulation of TLRs, which are crucial factors for the physiology of epithelial healing, combined with the alterations in gut barrier integrity and specific microbiota alterations (reduction of *Lactobacillus* and proliferation of *Enterobacteriaceae*) are well-known to be involved in the pathogenesis of anastomotic leakage [44]. Thus, these results could be valuable regarding the possible effect of perioperative antibiotics in anastomotic complications after bowel resection.

## 4. Materials and Methods

### 4.1. Ethics Statement

The experimental protocol was reviewed and approved by the Institutional Animal Care and Use Committee of the Institute of Animal Science at the Chinese Academy of Agricultural Science (IAS2020-85).

### 4.2. Mice and Antibiotics Treatment

In the present study, thirty 21-day-old female ICR mice purchased from School of Medicine, Peking University (Beijing, China) were housed in the animal facility of the Chinese Academy of Agricultural Sciences under a controlled condition of the lighting cycle with light from 8:30 a.m. to 8:30 p.m., the temperature at 20 ± 5 °C. After 3 days of acclimatization, mice were randomly divided into three groups (CON, LM1, and LM5, respectively) of 10 mice each. The mice in LM1 and LM5 were administered with 1 and 5 g/L lincomycin in drinking water for 1 week, respectively. Control mice received drinking water for 1 week. All mice had free access to food and drinking water, and water containers and feed were changed once three days to supply fresh antibiotics and feed. At the end of the experiment (week 1), body weight, food intake, and water intake were measured for mice, and the jejunum, ileum, colon, and colonic chyme were excised. All samples were either fixed in formalin or snap-frozen and stored at −80 °C until processing.

### 4.3. Cytokine Genes Expression

Total RNA was extracted using TRIzol reagent (Ambion, UT, USA). Total RNA was reversely transcribed into cDNA using a reverse transcription kit at 37 °C for 15 min, 85 °C 5 s. All primers were designed on the National Center for Biotechnology Information website (Table 2). The House-keeping gene was GADPH to normalize target gene levels. Quantitative real-time RT-PCR (qRT-PCR) was performed using TB Green Premix Ex Taq (TaKaRa, Kusatsu, Japan). The gene of tight junction protein (*Occludin*, *ZO-1*, and *Claudin-1*), pro-inflammatory cytokines (*TNF-α*, *IL-1β*, and *IL-18*), TLR (*TLR2*, *TLR3*, *TLR4*, and *TLR6*), *NF-κB*, and *P65* were analyzed. The primers used for q-PCR were purchased from Sangon Biotech (Shanghai, China) and shown in Table 2. All results of the target genes were normalized to the housekeeping gene GAPDH as an endogenous control. Reactions were run on an Applied Biosystems 7500 system (Applied Biosystems, Life Technologies, Waltham, MA, USA) using the following protocol: 94 °C for the 30 s, 40 cycles of 5 s at 94 °C, 15 s at 58 °C, 10 s at 72 °C. Results were analyzed using 2^−∆∆Ct^ methods.

### 4.4. DNA Extraction, 16S rRNA Gene Amplification and Sequencing

The E.Z.N.A.^®^ soil DNA Kit (Omega Bio-tek, Norcross, GA, USA) was used to extract microbial community genomic DNA on the basis of the manufacturer’s instructions. According to the method previously described, the hypervariable region V3-V4 of the bacterial 16S rRNA gene was amplified with primer pairs 338F (5’-ACTCCTACGGGAGGCAGCAG-3’) and 806R (5’-GGACTACHVGGGT WTCTAAT-3’) using an ABI Gene Amp^®^ 9700 PCR thermocycler (ABI, CA, USA) [45]. The meta-genomic sequencing was performed based on Illumina platform using Miseq PE300. The raw reads were deposited into the NCBI Sequence Read Archive (SRA) database (Accession Number: PRJNA665786).

### 4.5. Sequence Processing and Analysis

The raw sequences were processed using Majorbio I-Sanger Cloud Platform (www.i-sanger.com, Majorbio, Shanghai, China), and chimeric sequences were removed. Operational taxonomic units (OTUs) with a 97% similarity cutoff were clustered using UPARSE (version 7.1, http://drive5.com/uparse/). The taxonomy of each OTU representative sequences were matched to the 16S rRNA database (Silva SSU128) with a confidence threshold of 0.7 using RDP Classifier (http://rdp.cme.msu.edu/). Mothur b.1.30.1 [46] was used to perform refraction analysis, alpha-diversity analysis, and unweighted principal coordinate analysis (PCoA). Sobs, Shannon and Chao indices were employed to evaluate community diversity. For each group, a network based on spearman’s coefficients was established to depict the correlations among cytokine contents, the relative abundances of bacterial genera, and SCFA concentrations in colon.

### 4.6. The Relative Concentration of SCFAs

The colon chyme samples, approximately 0.1 g of each mouse, were suspended in 1 mL of ddH2O in a 1.5 mL screw-capped tube for SCFAs composition analysis. The gas chromatography method was used to determine the composition of SCFAs as Wu et al. [47].

### 4.7. Tissue Sample and Intestinal Morphology

Jejunum and ileal segments were immersed and fixed with formalin for 24 h to observe intestinal morphology, and then were cut into 5 μm-thick sections stained with hematoxylin and eosin (H&E) [48]. The villous height and crypt depth of each sample were measured and the villous height/crypt depth ratio was calculated.

### 4.8. Statistical Analysis

For the data of growth performance, intestinal morphology, SCFAs concentration, and mRNA expression, one-way ANOVA with multiple comparisons using Fisher LSD test was performed (SAS 9.4, Institute, Cary, NC, USA), accepting statistically significant at the level of *p*-value < 0.05.

## 5. Conclusions

In conclusion, this study indicated that a week of lincomycin exposure decreased the concentrations of SCFA, altered intestinal morphology, and reduced the elevated expression of TLRs and inflammation-related cytokines in infant mice via alterations of the gut microbiota. Furthermore, these effects differed for different doses of lincomycin. Due to the increased relative abundance of pathogenic bacteria and decreased relative abundance of beneficial bacteria, this might cause subsequent long-term negative impacts of lincomycin on host intestinal health in mice. The absence of follow-up evaluation of the restoration of gut microbiota is one major limitation of this study. Thus, the subsequent long-term evaluation of the restoration of gut microbiota worth be worth study in the future.

## Figures and Tables

**Table 1:** Effects of lincomycin on the morphology of jejunum and ileum in mice.

	Jejunum µm			Ileum µm		
Group	Villus Height	Crypt Depth	V/C	Villus Height	Crypt Depth	V/C
CON	470.26 ^a^	111.02 ^a^	4.29	209.46 ^a^	79.16 ^a^	2.63
LM1	405.51 ^ab^	95.24 ^ab^	4.23	161.12 ^ab^	65.47 ^ab^	2.45
LM5	310.30 ^b^	78.58 ^b^	4.02	137.75 ^b^	55.68 ^b^	2.46
SEM	21.37	4.35	0.15	12.12	3.09	0.10
*p*-value	0.006	0.007	0.897	0.048	0.004	0.470

^a,b^ Values in the same column not sharing a common superscript differ significantly (*p* < 0.05).

**Table 2:** Primers Used for q-PCR Analysis.

Gene	Forward Nucleotide Sequence Primers (5′-3′)	Reverse Nucleotide Sequence Primers (5′-3′)	Product Size (bp)
GADPH	ACCACAGTCCATGCCATCAC	TCCACCACCCTGTTGCTGTA	172
TLR2	AGGTGCCCTGTGCCACCATT	CGGAACGAAGTCCCGCTTGT	172
TLR3	GGTCCCCAGCCTTCAAAGAC	ACGAAGAGGGCGGAAAGGT	85
TLR4	CTGGTGGCTGTGGAGACAAA	ATTCCCTGAAAGGCTTGGTC	172
TLR6	TCATCTCAGCAAACACCGAGTATAGCG	CAACCTTATTGAATGTGACCCTCCAGC	249
IL-1β	TCGCAGCAGCACATCAACAAGAG	AGGTCCACGGGAAAGACACAGG	97
IL-18	AGACCTGGAATCAGACAACTTT	TCAGTCATATCCTCGAACACAG	117
TNF-α	GGACTAGCCAGGAGGGAGAACAG	GCCAGTGAGTGAAAGGGACAGAAC	103
NF-kB	CAAAGACAAAGAGGAAGTGCAA	GATGGAATGTAATCCCACCGTA	203
P65	TCGAGTCTCCATGCAGCTACGG	CGGTGGCGATCATCTGTGTCTG	93
ZO-1	CTGGTGAAGTCTCGGAAAAATG	CATCTCTTGCTGCCAAACTATC	97
Occludin	TGCTTCATCGCTTCCTTAGTAA	GGGTTCACTCCCATTATGTACA	155
Claudin-1-1	AGATACAGTGCAAAGTCTTCGA	CAGGATGCCAATTACCATCAAG	86
